# Zoonotic Spillover of a Canine-like Rotavirus A G3P[3] Strain in a Brazilian Child

**DOI:** 10.3390/tropicalmed11060144

**Published:** 2026-05-26

**Authors:** Vanessa Cristina Martins Silva, Lais Sampaio Azevedo, Raquel Guiducci, Adriana Luchs

**Affiliations:** 1 Virology Center, Adolfo Lutz Institute, Av. Dr Arnaldo, nº 355, São Paulo 01246-902, SP, Brazil; 2 Graduate Program in Sciences of the Center for Disease Control, Sao Paulo State Department of Health, São Paulo 01246-000, SP, Brazil

**Keywords:** genetic diversity, interspecies transmission, rotavirus, gastroenteritis, molecular epidemiology

## Abstract

Rotavirus A (RVA) G3P[3] genotype is widely reported in dogs and less frequently in cats, with only sporadic human cases worldwide. All reported human infections have occurred in children, suggesting increased susceptibility likely linked to close contact with pets and age-related hygiene practices. The identification of a novel genotype constellation in Brazilian canine G3P[3] strains in 2017 prompted full-genotype characterization of the historical RVA/Human-wt/BRA/IAL-R451/2011/G3P[3] strain, previously sequenced only for VP7 and VP4, to define its genomic constellation and relatedness to canine strains. All 11 segments were analyzed by RT-PCR, sequencing and phylogenetics. The rare genotype–lineage constellation G3.III-P[3]-I2.XX-R3.II-C2.V-M3.II-A9-N2.XXIV-T3.II-E3.II-H6.I, shared with Brazilian canine strains, was identified, supporting a potential common origin. RVA/Human-wt/BRA/IAL-R451/2011/G3P[3] strain showed high genetic similarity (93.2–99%) with canine, feline and canine/feline-like human strains worldwide, with six genes (VP1, VP6 and NSP2–NSP5) closely related to Brazilian dog isolates (97.6–99%), indicating its canine origin. NSP2 clustered with strains from domestic (bovine), synanthropic (rat) and human hosts, while VP7 and VP4 were associated with wildlife (bat; raccoon dog) and environmental (sewage; river water) strains, supporting interhost reassortment and highlighting aquatic environments as reservoirs for interspecies transmission. Identification of new lineages (VP1, VP3 and NSP2) within the AU-1-like backbone reflects its underexplored diversity. This novel constellation likely circulated in dogs and may spill over to humans via close contact, reinforcing a One Health approach to understand RVA zoonotic risk, especially in hotspot regions like Brazil.

## 1. Introduction

Rotavirus A (RVA), currently classified as *Rotavirus alphagastroenteritidis* [1], remains the leading viral agent responsible for acute gastroenteritis in infants and young children globally [2]. The substantial burden of disease caused by RVA has prompted large-scale public health interventions, particularly the implementation of vaccination programs worldwide [2]. By the end of 2024, RVA vaccines had been introduced into the National Immunization Programs (NIPs) of 131 countries, representing a major global strategy to reduce morbidity and mortality associated with RVA infections [3].

Brazil was among the countries that adopted RVA vaccination early, introducing the Rotarix^®^ vaccine into its national immunization schedule in 2006 [4]. Following vaccine implementation, significant public health benefits were observed. A substantial decrease in diarrheal disease burden among children under five was recorded between 2006 and 2018, with hospitalizations dropping by 52.5% (68.4 to 32.5 per 10,000 children) [4]. Concurrently, diarrheal mortality decreased across the country to an average of 9.8% per year, with more pronounced reductions in the Northeast, where annual declines reached 13.9% [4].

Beyond its relevance to human health, RVA is also recognized for its wide host range, infecting numerous animal species. This broad host distribution not only contributes to considerable economic losses in livestock production but also creates opportunities for interspecies transmission and viral evolution [5,6].

RVA is taxonomically classified in the genus *Rotavirus*, family *Reoviridae*, order *Reovirales*, subfamily *Sedoreovirinae* and realm *Riboviria* [7]. The viral genome is composed of 11 segments of double-stranded RNA (dsRNA), which collectively encode six structural proteins (VP1 through VP4, VP6 and VP7) and six non-structural proteins (NSP1 to NSP5/6) that play key roles in viral replication and assembly processes [8].

Traditionally, RVA strains have been identified using a binary genotyping system based on the two outer capsid proteins, VP7 and VP4, which define the G and P genotypes, respectively [9]. Among humans, the most frequently reported genotype combinations include G1P[8], G2P[4], G3P[8], G4P[8], G9P[8] and G12P[8] [10]. However, additional genotypes such as G5, G6, G8, G10, G11 and P[1], P[5], P[7], P[9] and P[14] have occasionally been detected in humans [11,12,13]. These strains are thought to arise from zoonotic transmission events, reflecting the capacity of RVA to undergo interspecies reassortment [11,12,13].

To provide greater resolution in the characterization of RVA strains, a whole-genome classification system was later introduced. This system assigns specific genotypes to each of the eleven genomic segments and is represented by the nomenclature Gx–P[x]–Ix–Rx–Cx–Mx–Ax–Nx–Tx–Ex–Hx [14]. Based on this genomic constellation, most human RVA strains are grouped into three major genogroups: Wa-like (Genogroup 1), DS-1-like (Genogroup 2) and AU-1-like (Genogroup 3), each defined by characteristic genotype constellations across the genome segments [14].

This classification system also provides a robust framework for inferring the evolutionary origin and genetic background of RVA strains. Full-genome genotypic characterization is particularly important for rare or unusual RVA strains, as it facilitates the detection of reassortment events, potential zoonotic origins and genetic relationships that cannot be identified through binary genotyping system [11,12,13].

The G3P[3] genotype is most frequently detected in dogs and, less commonly, in cats and other wild animals such as bats, rats and rabbits, indicating a broad host range and potential for interspecies transmission; nevertheless, only a limited number of human infections have been reported worldwide [15,16,17,18,19,20,21,22,23,24,25,26,27,28,29,30]. Notably, all human G3P[3] infections have occurred in children [16,17,18,19,20,31], suggesting increased susceptibility in this age group, potentially associated with close contact with pets and less developed hygiene practices [20]. In contrast, infections in adults may be underreported, as they are more likely to be mild or asymptomatic, possibly due to partial immunity acquired from previous exposures to RVA, which can reduce the likelihood of symptomatic disease [32]. Serological investigations targeting G3P[3] RVA-specific antibodies could help shed light on this issue [20].

Recently, the identification of a novel genotype constellation in Brazilian canine G3P[3] RVA strains highlighted the genetic diversity of RVA circulating in domestic animals and suggested that dogs may act as reservoirs of reassortant strains [28]. In this context, the previously reported RVA/Human-wt/BRA/IAL-R451/2011/G3P[3] strain, originally identified in 2011 in a one-year-old child with acute gastroenteritis in Brazil, represents a valuable and still underexplored isolate, as only the outer capsid genes VP7 and VP4 had previously been characterized [20]. Therefore, the aim of the present study was to perform the complete genotype characterization of this strain to determine its genomic constellation and evaluate whether it shares genetic similarities with recently described canine strains, thereby providing insights into possible reassortment events and interspecies transmission of RVA in Brazil.

## 2. Materials and Methods

### 2.1. Study Design and Sample

The RVA/Human-wt/BRA/IAL-R451/2011/G3P[3] strain analyzed in this study was selected as part of a broader investigation aimed at exploring the evolutionary dynamics of animal-origin RVA strains infecting humans in Brazil. The study comprised the whole-genotype characterization of 83 RVA-positive samples collected between 2007 and 2020. All procedures were conducted within projects approved by the Technical-Scientific Council (CTC) of the Adolfo Lutz Institute (protocols CTC 45-G/2014 and CTC 02-N/2021).

### 2.2. RNA Extraction and Amplification of RVA Genome Segments

Viral dsRNA was extracted from 10% (*v*/*v*) fecal suspensions using the QIAamp^®^ Viral RNA Mini Kit (QIAGEN, Valencia, CA, USA) according to the manufacturer’s protocol. All eleven RVA genome segments were amplified by reverse transcription PCR (RT-PCR) using in-house protocol previously described by Gouvea et al. [33]. Primers targeting the VP1 and VP3 genes were described by Ramani et al. [34], VP2 by Wang et al. [35], VP4 by Gentsch et al. [36], NSP1 by He et al. [21] and VP6, VP7 and NSP2-5 by Magagula et al. [37]. PCR amplicons were resolved on 1.5% GelRed™-stained agarose gels (Biotium, Fremont, CA, USA) alongside a 100 bp ladder and visualized using the L-PIX TOUCH gel documentation system (Loccus do Brasil Ltda., São Paulo, SP, Brazil).

### 2.3. Sequencing and Genotype Determination

Sequencing of PCR amplicons was performed using the BigDye™ Terminator v3.1 Cycle Sequencing Kit (Applied Biosystems, Foster City, CA, USA), employing the same primer sets used for RT-PCR amplification [21,34,35,36,37]. The sequencing reactions were run on an ABI 3500 Genetic Analyzer (Applied Biosystems) at the Premium Network of Multi-User Equipment of the Institute of Tropical Medicine, University of São Paulo (IMT/FMUSP). Resulting chromatograms were edited and assembled using Sequencher™ v4.7 software (Gene Codes Corporation, Ann Arbor, MI, USA), and RVA genotypes were determined using the Rotavirus A Genotyping Tool v0.1 (https://mpf.rivm.nl/mpf/typingtool/rotavirusa/, accessed on 26 February 2025).

### 2.4. Sequence Analysis and Phylogenetic Inference

The nucleotide sequences obtained were aligned with representative RVA reference strains from GenBank, encompassing all genome segments (NSP1–NSP5, VP1–VP4, VP6–VP7), using the CLUSTALW algorithm in BioEdit v7.0.5.2 (Ibis Therapeutics, Carlsbad, CA, USA). Alignments comprised 992 nt for VP7, 762 nt for VP4, 1308 nt for VP6, 1061 nt for VP1, 718 nt for VP2, 466 nt for VP3, 1497 nt for NSP1, 971 nt for NSP2, 953 nt for NSP3, 684 nt for NSP4 and 667 nt for NSP5/6.

Phylogenetic relationships were inferred using the maximum likelihood (ML) method implemented in MEGA 12 (Molecular Evolutionary Genetics Analysis version 12, available for download at https://www.megasoftware.net/, accessed on 11 May 2026) [38]. Nucleotide substitution models for each gene segment were selected according to the corrected Akaike Information Criterion (AICc): General Time Reversible (GTR) + G + I for NSP1 (A9); Tamura 3-parameter (T92) + G + I for NSP2 (N2), NSP4 (E3), VP1 (R3), VP3 (M3), VP6 (I2) and VP7 (G3); T92 + G for NSP3 (T3), NSP5 (H6) and VP4 (P[3]); Tamura-Nei (TN93) + G + I for VP2 (C2). Statistical support for phylogenetic branches was evaluated based on 1000 bootstrap replicates. Pairwise nucleotide identity values were calculated using distance matrices produced in MEGA 12 [38].

**Figure 1:**
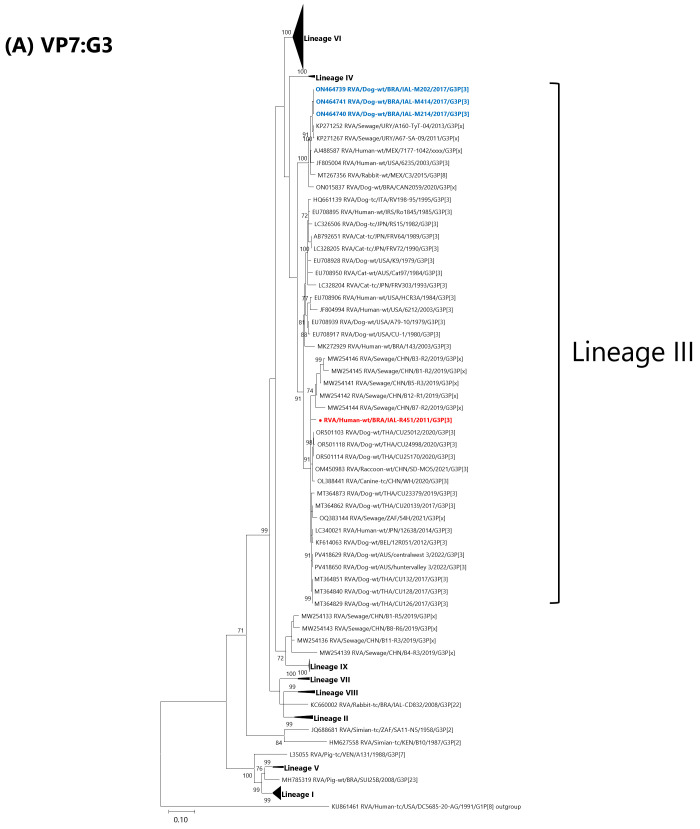

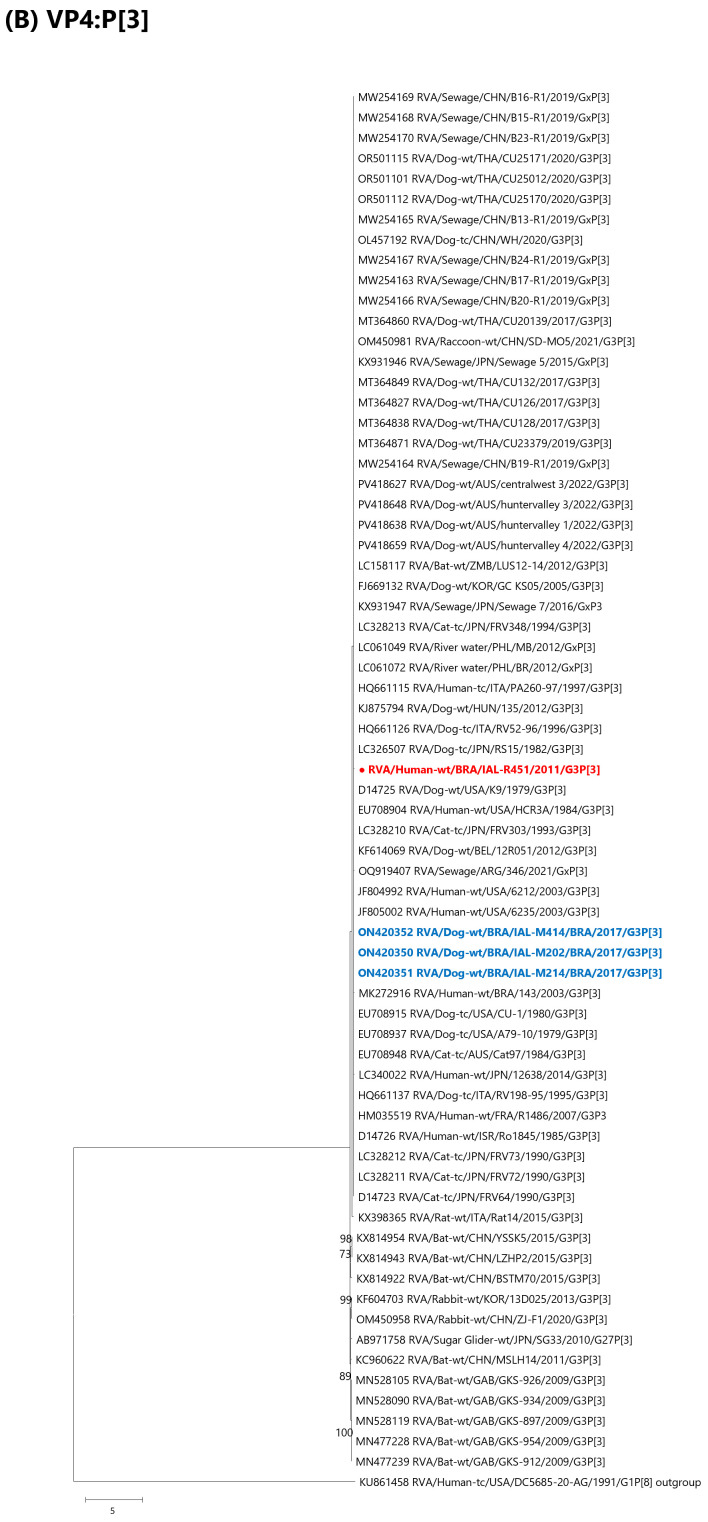

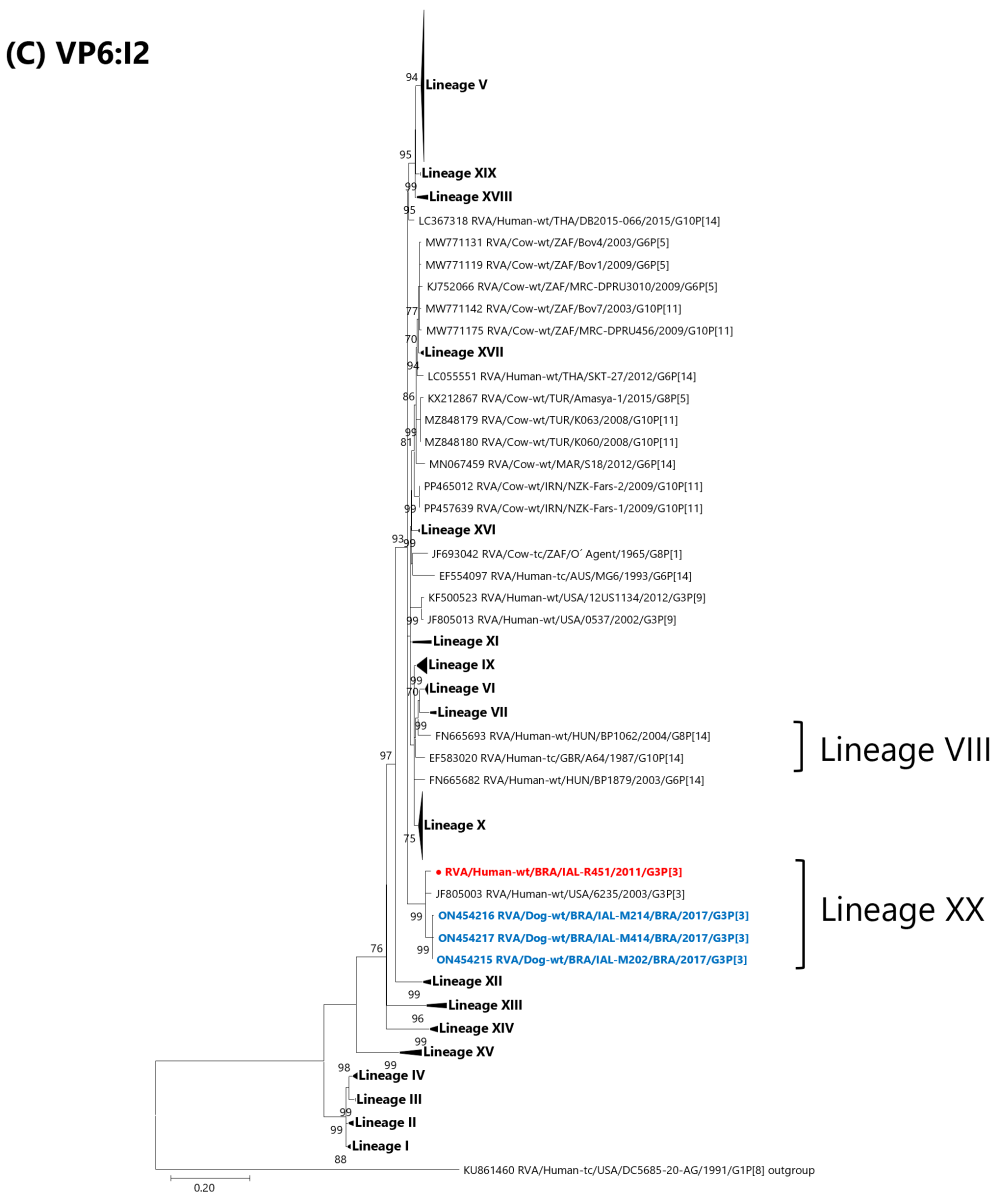

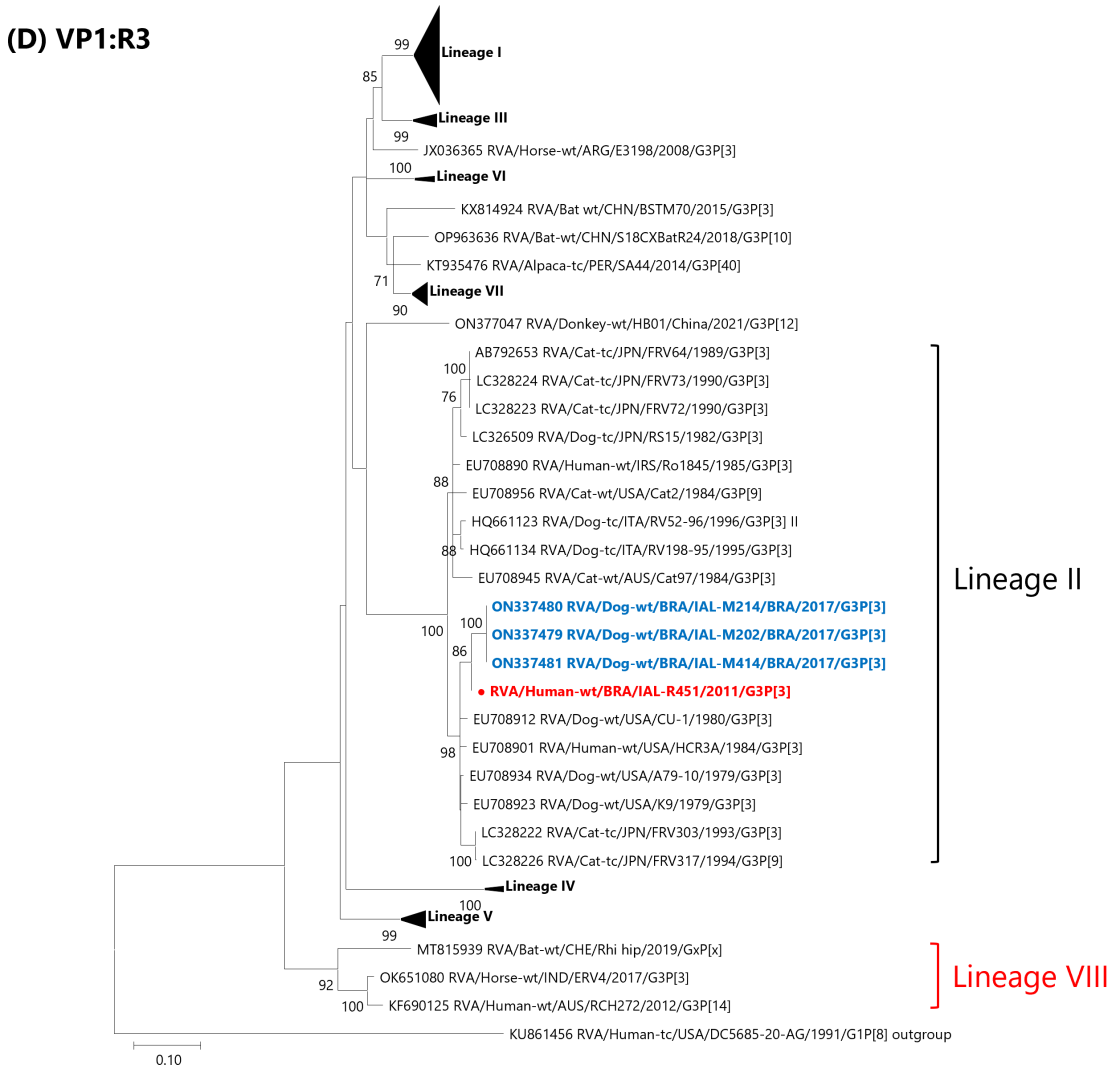

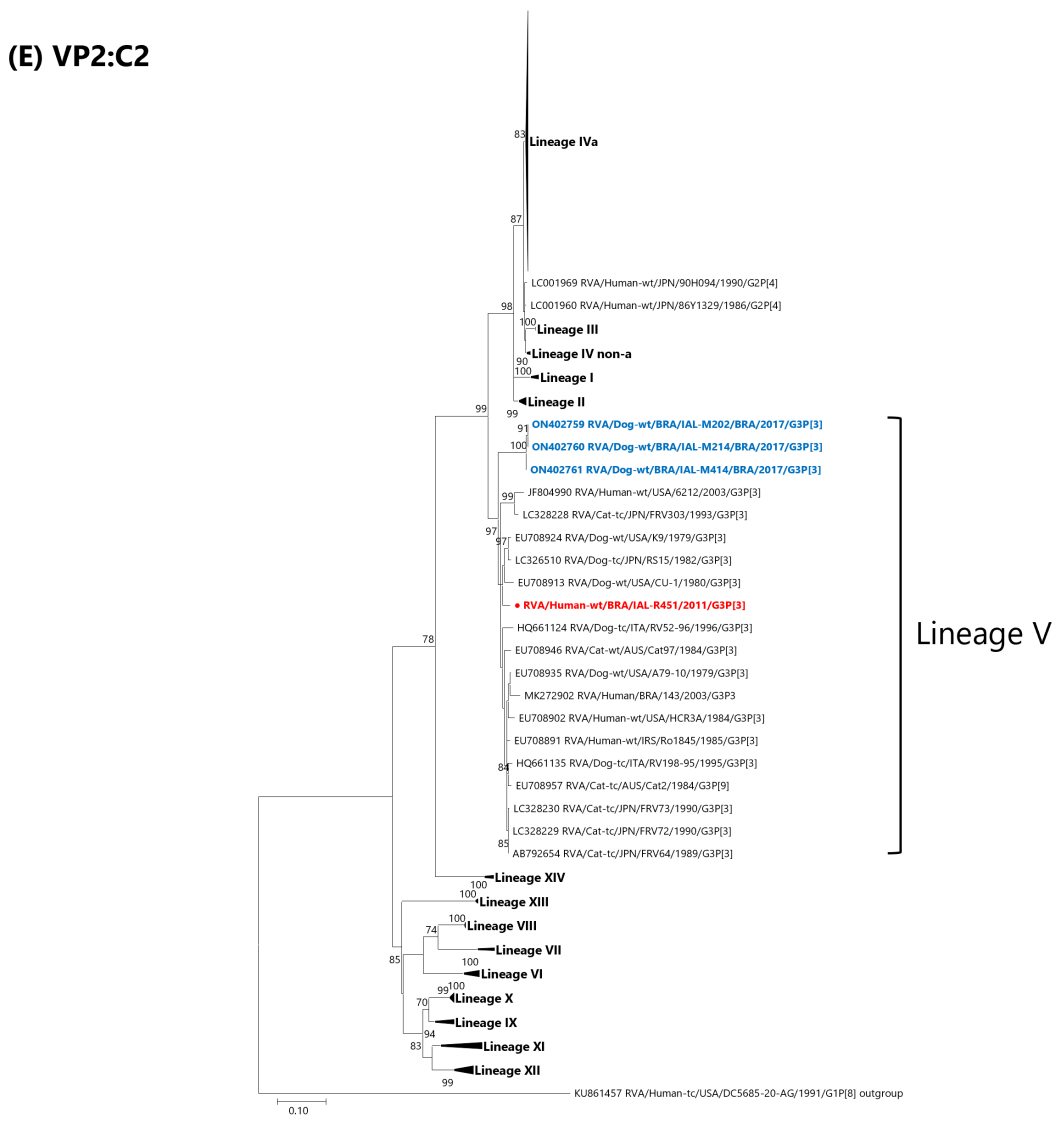

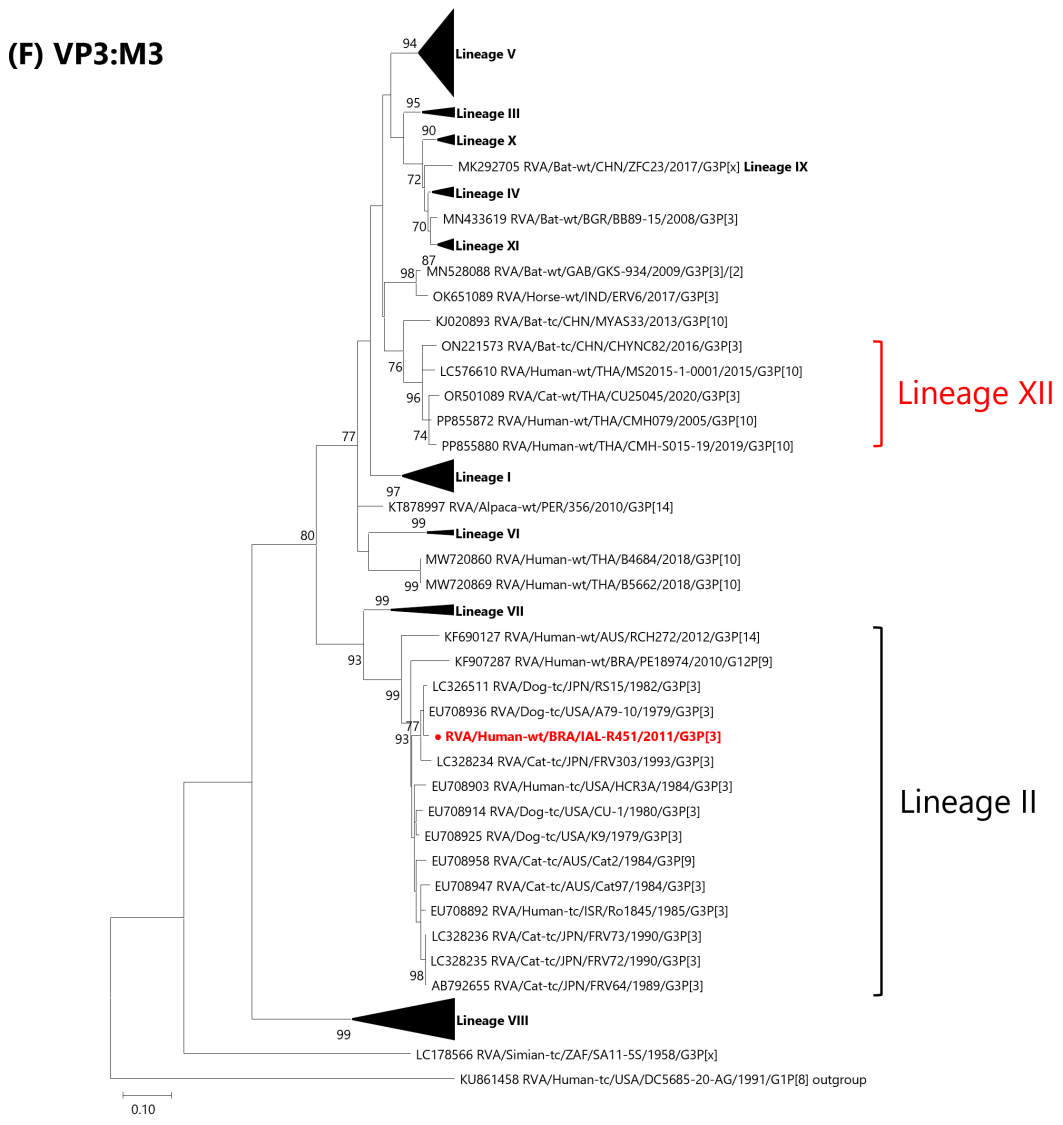

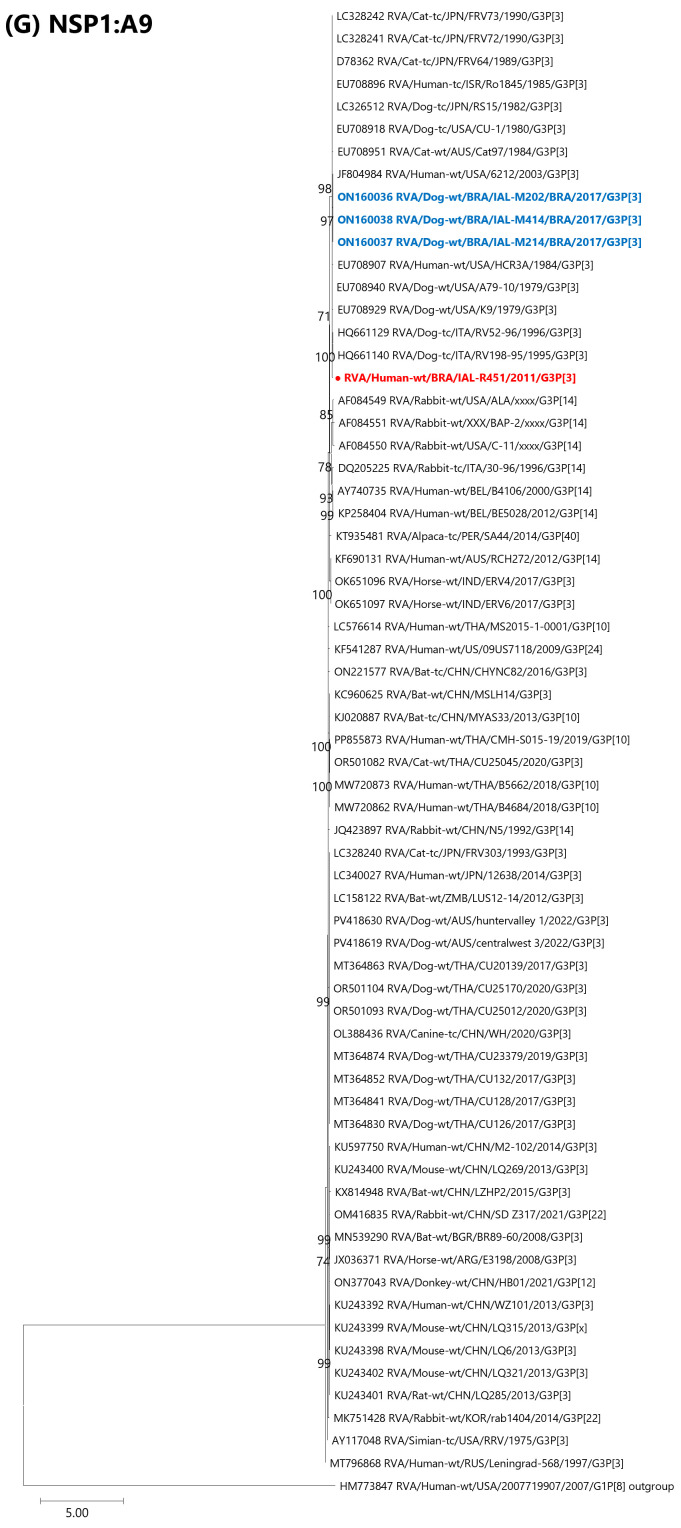

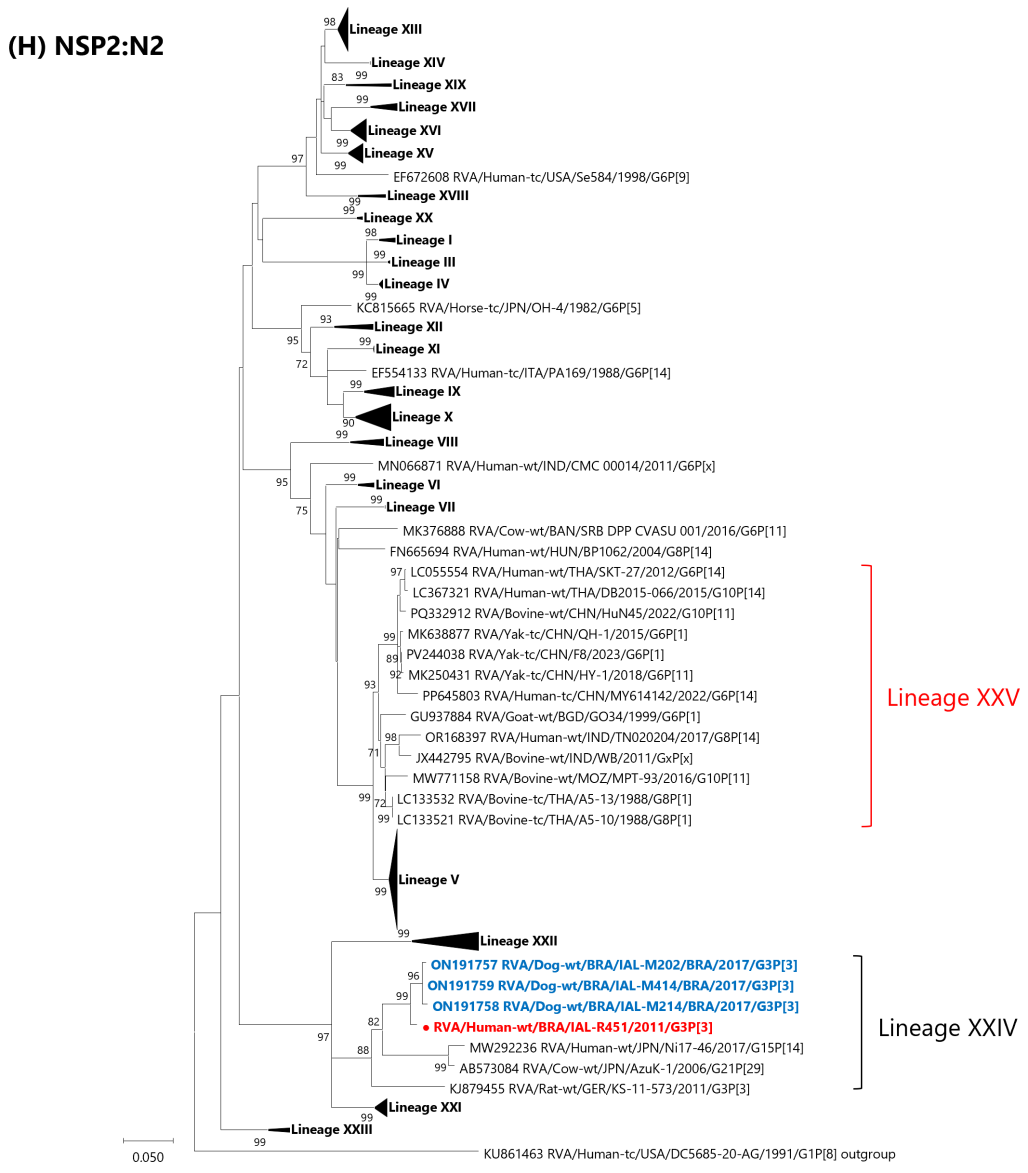

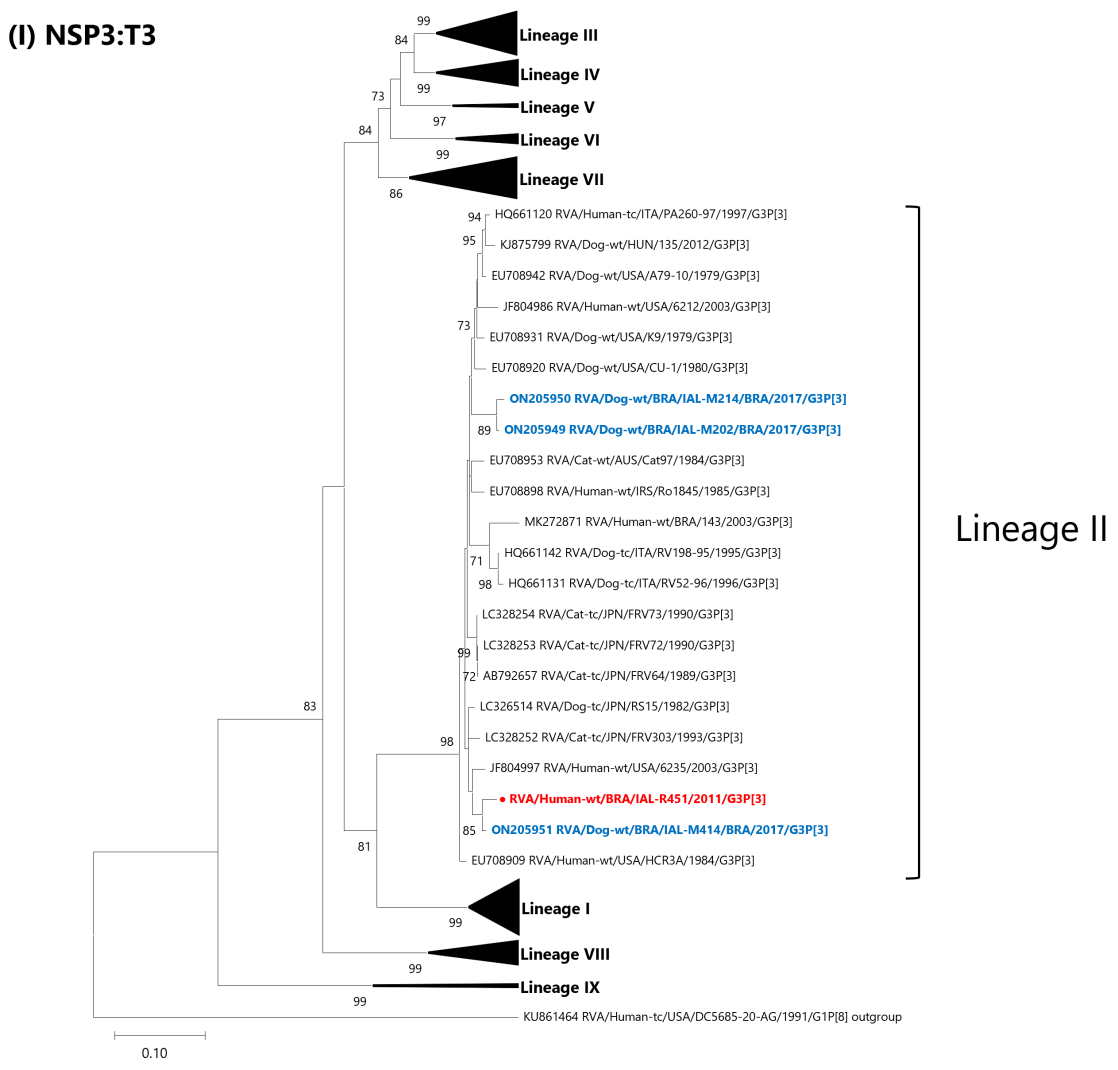

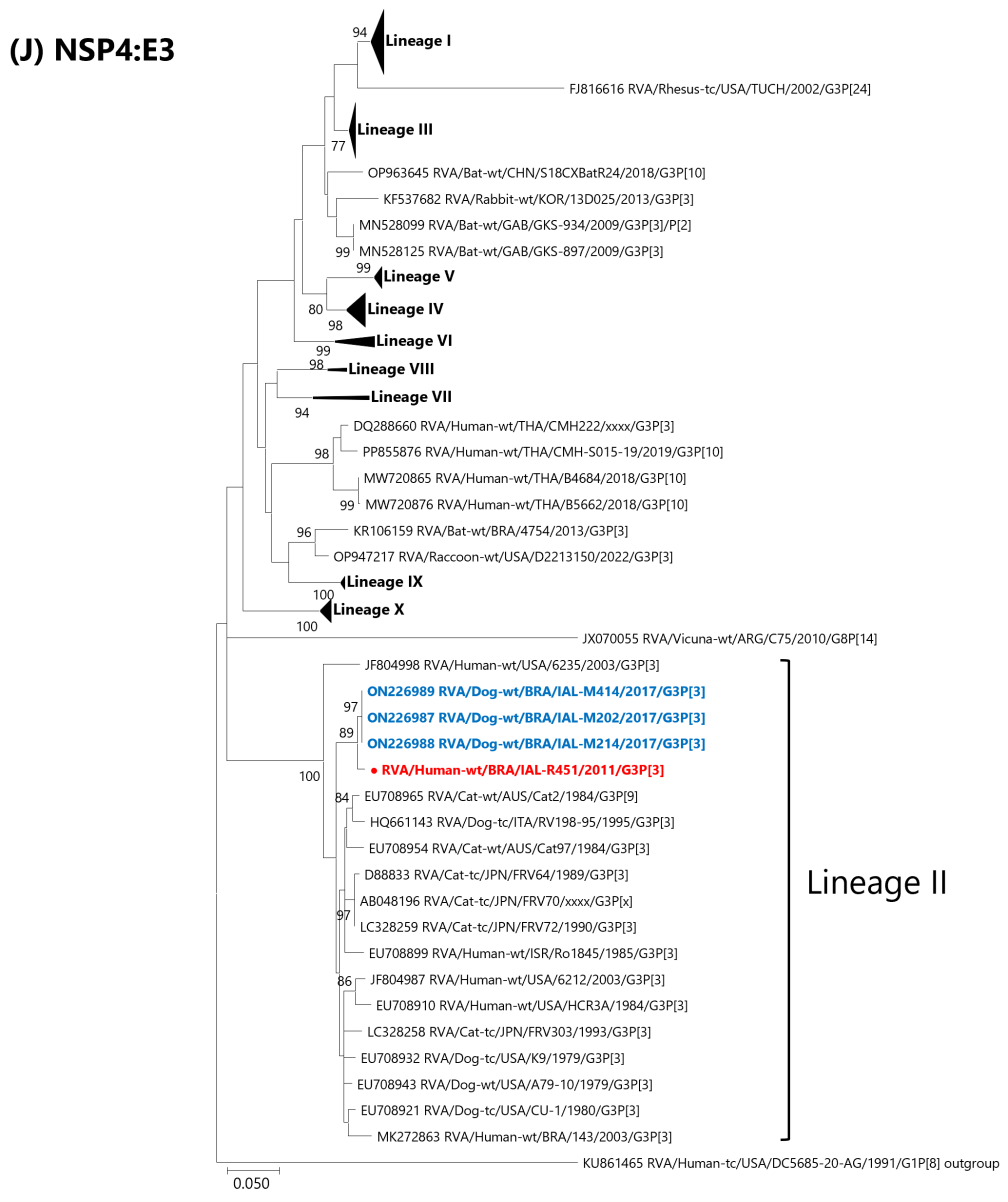

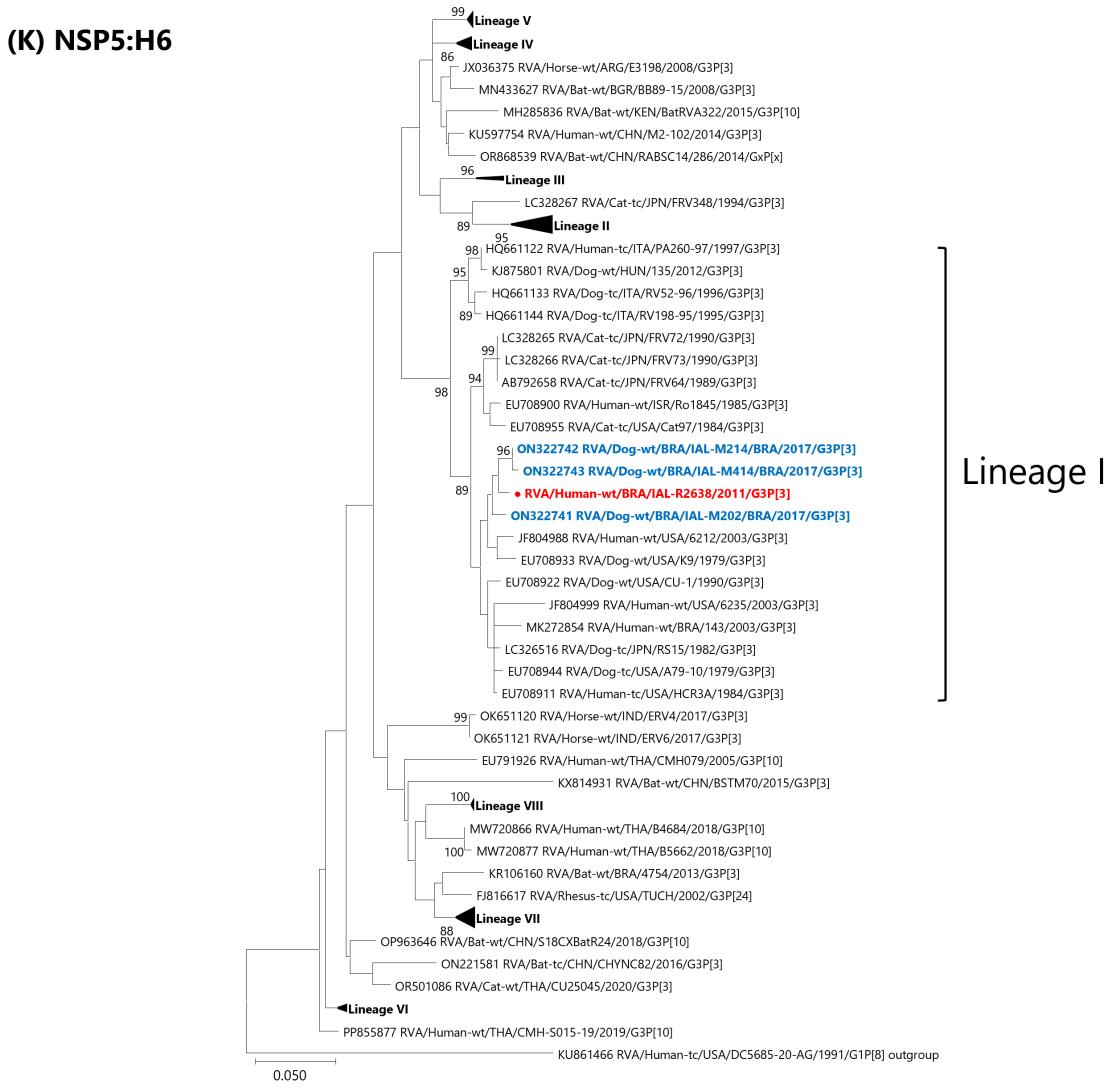
Phylogenetic relationships of selected rotavirus A (RVA) strains inferred from nucleotide sequences of individual genome segments. The Brazilian strain RVA/Human-wt/BRA/IAL-R451/2011/G3P[3] is highlighted in red (bold) and marked with a solid circle (●). Brazilian canine strains RVA/Dog-wt/BRA/IAL-M202/2017/G3P[3], RVA/Dog-wt/BRA/IAL-M214/2017/G3P[3] and RVA/Dog-wt/BRA/IAL-M414/2017/G3P[3] are indicated in bold dark blue. Phylogenetic trees were reconstructed using the maximum-likelihood method implemented in MEGA version 12. Separate analyses were performed for each genome segment, including (**A**) VP7, (**B**) VP4, (**C**) VP6, (**D**) VP1, (**E**) VP2, (**F**) VP3, (**G**) NSP1, (**H**) NSP2, (**I**) NSP3, (**J**) NSP4 and (**K**) NSP5/6. Alignments comprised 992 nt for VP7, 762 nt for VP4, 1308 nt for VP6, 1061 nt for VP1, 718 nt for VP2, 466 nt for VP3, 1497 nt for NSP1, 971 nt for NSP2, 953 nt for NSP3, 684 nt for NSP4 and 667 nt for NSP5/6. Reference sequences were retrieved from the GenBank database. For each strain included in the analysis, the genotype, lineage designation, accession number, isolate name, country of origin and year of detection are indicated in the trees. Putative novel lineages are highlighted in red. Branch lengths correspond to the number of nucleotide substitutions per site, and bootstrap support values are displayed at the corresponding nodes. Complete strain list in Table S1.

Reference sequences were included to determine lineage classification according to previously established criteria. Lineage-level analysis provides finer resolution than genotype-based classification alone, enabling the identification of closer evolutionary relationships among strains, more precise tracking of interspecies transmission events, and the detection of potential reassortment events. Lineages are denoted by Roman numerals. For the VP7 G3 genotype, lineage assignment followed the classification proposed by Katz et al. [39], while classifications for NSP2 (N2), VP2 (C2) and VP6 (I2) followed Agbemabiese et al. [40] and Azevedo et al. [28]. Lineage criteria for NSP3 (T3), NSP4 (E3), NSP5 (H6), VP1 (R3) and VP3 (M3) were based on Gauchan et al. [41] and Azevedo et al. [28]. Potential novel lineages suggested in this study (highlighted in red in Figure 1) within a genotype were considered significant when the bootstrap value at the branching point was ≥90%.

## 3. Results

Genome sequencing of the RVA/Human-wt/BRA/IAL-R451/2011/G3P[3] strain generated near-complete sequences for the NSP1–NSP5/6, VP6, and VP7 segments, whereas only partial sequences were obtained for the VP1-4 genes. The sequence lengths and nucleotide positions are shown in Supplementary File S1. Based on whole-genotype analysis, the strain was classified as G3-P[3]-I2-R3-C2-M3-A9-N2-T3-E3-H6, a constellation rarely identified in human RVA strains [19] and recently identified in Brazilian dogs [28]. In fact, the RVA/Human-wt/BRA/IAL-R451/2011/G3P[3] strain shares the same genotype lineage constellation as Brazilian canine RVA strains, defined as G3.III-P[3]-I2.XX-R3.II-C2.V-M3.II-A9-N2.XXIV-T3.II-E3.II-H6.I, further supporting their close genetic relationship and a possible common origin. A comparison with reference animal and animal-like human strains is presented in Table 1.

Phylogenetic and sequence analyses of the outer capsid genes revealed that the VP7 gene clustered within lineage III and showed the highest nucleotide identity (nt) with the canine strain RVA/Dog-wt/BEL/12R051/2012/G3P[3] (98.4%) [42]. Comparisons with previously reported Brazilian canine strains (IAL-M202, IAL-M214 and IAL-M414) showed identities ranging from 90.3% to 90.6%, while other canine strains displayed similarities between 90.1% and 97.5%. Nucleotide identities ranged from 93.6% to 95.4% with feline strains and from 89.5% to 97.8% with animal-like human strains, reaching 92.5% identity with the Brazilian strain RVA/Human-wt/BRA/143/2003/G3P[3]. High similarity was also observed with the wildlife strain RVA/Raccoon-wt/CHN/SD-MO5/2021/G3P[3] (97.1%) [27], whereas lower similarity was observed with the domestic animal strain RVA/Rabbit-wt/MEX/C3/2015/G3P[8] (89.0%) [43]. Environmental RVA sequences detected in sewage samples from China, South Africa and Uruguay shared 88.8–95.7% nucleotide identity (Figure 1A, Table S1).

The VP4 gene showed the highest nucleotide identity (94.5%) with the canine/feline-like human strain RVA/Human-tc/ITA/PA260-97/1997/G3P[3] and the feline strain RVA/Cat-tc/JPN/FRV348/1994/G3P[3], as previously reported [44,45]. Comparisons with additional feline strains showed nucleotide identities ranging from 92.1% to 93.6%, whereas canine/feline-like human strains displayed identities between 90.6% and 94.0%, including 91.5% identity with RVA/Human-wt/BRA/143/2003/G3P[3]. Canine strains shared 91.6–94.2% nucleotide identity overall, with slightly lower similarities observed with previously reported Brazilian canine strains (IAL-M202, IAL-M214 and IAL-M414), which ranged from 91.5% to 92.0% [28]. Environmental RVA sequences detected in sewage samples from Japan, Argentina, China and the Philippines exhibited nucleotide identities ranging from 90.5% to 93.4%, while a strain identified in river water in the Philippines showed 91.2% identity. Finally, strains identified in wild animals, such as bats (RVA/Bat-wt/ZMB/LUS12-14/2012/G3P[3]) and raccoons (RVA/Raccoon-wt/CHN/SD-MO5/2021/G3P[3]), displayed nucleotide similarities ranging from 92.4% to 97.7% (Figure 1B).

The VP6 gene showed the highest nucleotide identity (97.6%) with the canine-like human strain RVA/Human-wt/USA/6235/2003/G3P[3] [19]. It was also closely related to Brazilian canine strains RVA/Dog-wt/BRA/IAL-M202, RVA/Dog-wt/BRA/IAL-M214, and RVA/Dog-wt/BRA/IAL-M414 (96.9–97.2% nt) [28]. In the phylogenetic tree, these strains clustered within lineage XX (Figure 1C, Table S1).

Similarly, VP1 gene analysis showed that RVA/Human-wt/BRA/IAL-R451/2011/G3P[3] was most closely related (97.9% nt) to the canine strain RVA/Dog-wt/USA/A79-10/1979/G3P[3] and to the Brazilian canine strains RVA/Dog-wt/BRA/IAL-M202, RVA/Dog-wt/BRA/IAL-M214 and RVA/Dog-wt/BRA/IAL-M414 (97.9% nt) [18,28]. These strains clustered together with canine, feline and canine/feline-like human strains reported worldwide, sharing nucleotide identities ranging from 93.5% to 97.6% inside Lineage II. In addition, phylogenetic reconstruction of the VP1 gene revealed another R3 lineage, here designated lineage VIII, comprising strains detected in bats (RVA/Bat-wt/CHE/Rhi hip/2019/GxP[x]) [46], horses (RVA/Horse-wt/IND/ERV4/2017/G3P[3]) [47] and humans (RVA/Human-wt/AUS/RCH272/2012/G3P[14]) [48] (Figure 1D, Table S1).

Phylogenetic analysis based on the VP2 gene indicated that RVA/Human-wt/BRA/IAL-R451/2011/G3P[3] belonged to lineage V, sharing the highest nucleotide identity (97.7%) with the feline strain RVA/Cat-tc/AUS/Cat2/1984/G3P[9] [18]. The strain also shared 95.4–95.8% nucleotide identity with the Brazilian canine strains RVA/Dog-wt/BRA/IAL-M202, RVA/Dog-wt/BRA/IAL-M214 and RVA/Dog-wt/BRA/IAL-M414 [28]. Overall, the VP2 gene shared 94.4% to 97.4% nucleotide identity with other G3P[3] isolates from cats and dogs, as well as with canine/feline-like human strains (95.9% nt identity with RVA/Human-wt/BRA/143/2003/G3P[3]) (Figure 1E, Table S1).

The VP3 gene of RVA/Human-wt/BRA/IAL-R451/2011/G3P[3] showed the highest nucleotide similarity with the canine strain RVA/Dog-wt/USA/A79-10/1979/G3P[3] (98.9%), followed by RVA/Dog-tc/JPN/RS15/1982/G3P[3] (98.3%) [18]. In the phylogenetic tree, this strain clustered with other feline, canine and animal-like human strains within lineage II. The Brazilian canine strains RVA/Dog-wt/BRA/IAL-M202, RVA/Dog-wt/BRA/IAL-M214 and RVA/Dog-wt/BRA/IAL-M414 could not be included in the investigation, as only partial VP3 sequences were available for these strains, corresponding to a genomic region distinct from that amplified in the present study, impairing any phylogenetic analysis. In addition, the VP3 phylogenetic tree revealed one additional M3 lineage, here designated lineage XII, supported by a robust bootstrap value of 96%. This lineage comprised three human strains and one feline strain detected in Thailand, as well as one bat strain identified in China (Figure 1F, Table S1).

The NSP1 gene of the RVA/Human-wt/BRA/IAL-R451/2011/G3P[3] strain belonged to the A9 genotype and shared its highest nucleotide identity with the canine-like human strain RVA/Human-tc/ISR/Ro1845/1985/G3P[3] (93.2% nt) [18]. Comparisons with the Brazilian canine G3P[3] strains RVA/Dog-wt/BRA/IAL-M202/2017, RVA/Dog-wt/BRA/IAL-M214/2017 and RVA/Dog-wt/BRA/IAL-M414/2017 revealed slightly lower identities, ranging from 90.8% to 91.2% [28]. Similar levels of relatedness were observed with other canine, feline, and canine/feline-like human G3P[3] strains worldwide (91.5–92.6% nt) (Figure 1G).

Phylogenetic reconstruction of the NSP2 gene placed the Brazilian human strain within lineage XXIV, where it clustered closely with the Brazilian canine strains IAL-M202, IAL-M214 and IAL-M414, sharing high nucleotide identities (97.7–98.4%). Additional members of this lineage included RVA/Cow-wt/JPN/AzuK-1/2006/G21P[29], RVA/Human-wt/JPN/Ni17-46/2017/G15P[14] and RVA/Rat-wt/GER/KS-11-573/2011/G3P[3], which displayed more moderate similarities (≈90–91% nt) [49,50,51]. RVA/Human-wt/BRA/143/2003/G3P[3] clusters in a distinct lineage from RVA/Human-wt/BRA/IAL-R451/2011/G3P[3] (lineage XXII), sharing only 84.4% nucleotide similarity. A novel cluster, proposed as lineage XXV, comprised mainly bovine RVA strains from Asia and Africa, along with yak, goat and several human strains (bootstrap support of 93%) [52,53,54,55,56,57] (Figure 1H, Table S1).

Analysis of the NSP3 gene indicated the closest relationship with the Brazilian canine strain RVA/Dog-wt/BRA/IAL-M414/2017/G3P[3] (98.0% nt) [28]. Slightly lower nucleotide identities were observed with the canine strain RVA/Dog-tc/JPN/RS15/1982/G3P[3] (96.2% nt) and the canine/feline-like human strains RVA/Human-wt/USA/6235/2003/G3P[3] and RVA/Human-wt/USA/HCR3A/1984/G3P[3] (96.1–96.2% nt) [18,19,45]. The remaining Brazilian canine strains, IAL-M202 and IAL-M214, were somewhat more divergent (93.6–93.8% nt), as well as the canine-like human Brazilian strain RVA/Human-wt/BRA/143/2003/G3P[3] (92.9% nt). Phylogenetically, all sequences were grouped within lineage II (Figure 1I, Table S1).

For the NSP4 gene, the Brazilian human strain exhibited very high similarity to the Brazilian canine G3P[3] strains IAL-M202, IAL-M214 and IAL-M414 (98.9–99.0% nt), all assigned to lineage II [28]. More distant relationships were observed with other canine, feline and canine/feline-like human strains circulating globally, including the Brazilian strain RVA/Human-wt/BRA/143/2003/G3P[3], with nucleotide identities ranging from 95.1% to 97.1% (Figure 1J, Table S1).

Similarly, the NSP5 gene displayed its highest nucleotide similarity to the Brazilian canine strains IAL-M202, IAL-M214, and IAL-M414 (98.4–98.8% nt) [28]. In the phylogenetic tree, the Brazilian human strain clustered within lineage I, together with classical canine, feline, and canine/feline-like human RVA strains, sharing nucleotide identities between 95.5% and 97.7% (Figure 1K, Table S1).

## 4. Discussion

Sequencing data on human G3P[3] RVA strains remain extremely limited, particularly with respect to full-genotype characterization. To date, complete genotype constellation data are available in GenBank for just seven strains, originating from Japan, Israel, Russia, Italy and the United States [18,19,44,58]. From Brazil, only a single human G3P[3] RVA strain has been deposited in GenBank; however, this sequence is incomplete, lacking three genomic segments (VP1, VP3 and NSP1) [31]. The present study provides the first complete genotype characterization of a human G3P[3] strain detected in Brazil. Such comprehensive genomic analyses are essential for tracing the origin of unusual RVA strains and enhancing our understanding of their evolutionary dynamics.

Consistent with previous reports of human G3P[3] RVA strains worldwide, phylogenetic analyses of the RVA/Human-wt/BRA/IAL-R451/2011/G3P[3] strain revealed close genetic relationships with canine, feline and canine/feline-like human RVA strains, clustering within typical animal-associated lineages [18,19,44,58]. Furthermore, this strain exhibits a predominantly canine genetic backbone, sharing an identical genotype constellation (G3-III-P[3]-I2-XX-R3-II-C2-V-M3-II-A9-N2-XXIV-T3-II-E3-II-H6-I) with Brazilian canine G3P[3] strains reported in 2017 [28]. Brazilian canine G3P[3] IAL-M202, IAL-M214 and IAL-M414 strains have been described as representing a potential novel canine RVA genetic constellation, characterized by the VP6 I2 genotype [28], supporting a close genetic relationship and a possible common origin. A similar genotype constellation was previously reported for the RVA/Human-wt/USA/6235/2003/G3P[3] strain, detected in American Indian children in the United States during a phase III RVA vaccine clinical trial in 2003 [19]. However, it is important to note that the VP2 gene of the 6235/2003/USA strain could not be genotyped, preventing full constellation assignment [19]. Of note, the other canine/feline-like human strain identified in Brazil, RVA/Human-wt/BRA/143/2003/G3P[3] [31], does not possess the VP6 I2 genotype and instead harbors the VP6 I3 genotype, which is classically associated with canine RVA strains worldwide [22,25,29,30], thereby underscoring the genomic heterogeneity observed among zoonotic-like Brazilian human G3P[3] strains.

A more in-depth phylogenetic analysis of the RVA/Human-wt/BRA/IAL-R451/2011/G3P[3] strain in comparison with Brazilian canine strains (IAL-M202, IAL-M214 and IAL-M414) demonstrated high genetic relatedness across six genomic segments (VP1, VP6 and NSP2–NSP5). The NSP2 gene (genotype N2) clustered within lineage XXIV, together with homologous sequences from domestic (bovine), synanthropic (rat) and human RVA strains from Japan and Germany [49,50,51], consistent with the pattern previously reported for Brazilian canine IAL strains [28]. This clustering corroborates a shared evolutionary origin for this genomic segment. In addition, the VP7 (genotype G3) and VP4 (genotype P[3]) genes showed close relationships with strains derived from wild animals, such as bats [23] and raccoon dogs [27]. Altogether, these data suggest the involvement of reassortment events across multiple host species. The VP6 gene formed a distinct and well-supported cluster (lineage XX) comprising exclusively Brazilian canine strains (IAL-M202, IAL-M214 and IAL-M414) and canine/feline-like human strains, including RVA/Human-wt/BRA/IAL-R451/2011/G3P[3] and RVA/Human-wt/USA/6235/2003/G3P[3] [19], further reinforcing the hypothesis that these human strains may have originated from canine RVA lineages.

These findings highlight the genetic connectivity between human and animal RVA strains and underscore the potential role of interspecies transmission and reassortment in shaping the genomic constellation of G3P[3] strains. Interspecies transmission and reassortment between human and animal RVA are key mechanisms driving viral evolution [6,44]. This dynamic is driven by the segmented RVA genome, which enables reassortment during co-infection at the human–animal interface, potentially generating novel genotype constellations that are partially or fully compatible with human hosts [59]. In this context, the increasingly close coexistence between humans and companion animals further expands opportunities for cross-species transmission. The outer capsid proteins VP4 and VP7 are considered central to this process, mediating host cell attachment, entry and antigenic specificity [8,9,32]. In fact, among G genotypes, G3 stands out for its broad host range, infecting humans and multiple animal species [15,16,17,18,19,20,21,22,23,24,25,26,27,28,29,30], a feature that may favor human infection by canine-origin G3 strains. The recent global emergence of atypical G3 strains arising from human–animal reassortment, including the equine-like G3P[8] DS-1-like strains, also reported in Brazil, supports this hypothesis and highlights the role of zoonotic contributions in RVA evolution [19,39,60]. However, spillover success is not determined solely by outer capsid proteins; the genomic backbone, including structural and non-structural proteins, is also critical for viral fitness, replication and host adaptation [59]. Therefore, genome constellation compatibility likely determines whether reassortant strains can infect humans and sustain transmission. In this regard, comprehensive genomic analyses of globally identified RVA G3P[3] strains, including those from Brazil, are crucial to elucidate their evolutionary origins and assess their potential as emerging human pathogens.

A key challenge in interpreting zoonotic-like RVA strains is distinguishing between isolated interspecies transmission events and those capable of sustained human-to-human spread, as not all spillover infections result in epidemiologically relevant transmission. In this context, the RVA/Human-wt/BRA/IAL-R451/2011/G3P[3] strain may represent a “dead-end” spillover event [61]. Its high genomic similarity to canine strains, combined with the absence of evidence for secondary cases, supports this interpretation. Conversely, the occurrence of reassortment events involving multiple host species may facilitate further adaptation. In addition, the detection of related G3P[3] strains in different geographical settings suggests that some animal-derived strains may occasionally overcome host barriers and circulate at low frequency in humans. Overall, the evolution of G3P[3] strains likely represents a continuum from transient spillovers to variants capable of sustained transmission.

The detection of genetic relatedness with RVA VP7 (G3) and VP4 (P[3]) strains identified in environmental samples, including sewage and river water, emphasizes the role of aquatic environments as reservoirs for RVA strains. Previous studies have demonstrated a high prevalence of RVA in water matrices, particularly in untreated sewage, and highlighted their contribution to viral persistence and dissemination [62,63]. Moreover, environmental pathways may facilitate RVA spread between communities and across species, acting as intermediaries in transmission cycles at the human–animal–environment interface.

Potential new lineages are suggested for the VP1 (R3), VP3 (M3), and NSP2 (N2) gene segments. Ongoing refinement of lineage classification is crucial for enhancing the resolution of evolutionary relationships among RVA strains circulating worldwide [39,40]. Increased lineage diversity has been observed in gene segments shared between human and animal RVA strains, such as NSP2 (N2), likely reflecting their expanded host range and enhanced adaptive potential [28,40]. Even so, the increasing number of newly described lineages within AU-1-like backbone constellations (genogroup 3) highlights the dynamic nature of these strains and underscores the significant role of interspecies transmission and reassortment events in driving RVA evolution [28]. Integrated human–animal genomic surveillance is crucial for understanding RVA emergence, diversification, and zoonotic potential.

A limitation of the present study is the absence of detailed clinical and epidemiological information for the patient, including potential exposure to pets or other animals and RVA vaccination status. This constraint hampers a more comprehensive evaluation of potential risk factors and transmission pathways. Currently, inferences regarding zoonotic transmission of RVA G3P[3] strains rely largely on phylogenetic and genomic evidence, highlighting the importance of integrating molecular data with robust epidemiological information to strengthen conclusions on interspecies transmission dynamics [18,20]. Although vaccination data were not available, the identification of an uncommon genotype constellation raises questions about the potential influence of vaccine-driven selective pressures on the emergence or detection of atypical RVA strains. Continued surveillance will be essential to determine whether such strains could impact vaccine effectiveness. Another limitation of this study was the inability to obtain complete sequences for the VP1–VP4 genes, which hindered more comprehensive genetic comparisons and limited the strength of the evolutionary inferences. This limitation is particularly relevant for the VP3 segment, as Brazilian canine strains could not be incorporated into the phylogenetic analysis due to the lack of overlapping genomic regions. To mitigate this limitation, future investigations should consider obtaining full-length sequences using next-generation sequencing (NGS) approaches. Nevertheless, successful recovery of complete genomes using NGS is contingent upon the integrity of the viral RNA, which may be compromised in archival samples subjected to long-term storage. This constraint is particularly relevant for the RVA/Human-wt/BRA/IAL-R451/2011/G3P[3] strain analysed in this study, originally detected approximately 15 years prior.

## 5. Conclusions

In conclusion, these findings suggest that the G3-P[3]-I2-R3-C2-M3-A9-N2-T3-E3-H6 genomic constellation has likely circulated in canine populations for decades, with potential spillover into humans, particularly children, through close contact with companion animals. Although the genomic backbone of the RVA/Human-wt/BRA/IAL-R451/2011/G3P[3] strain is predominantly canine-like, its consistent phylogenetic association with wildlife, feline, bovine, rodent, and human RVA strains across multiple genomic segments indicates a complex evolutionary history shaped by frequent interspecies transmission events. This finding reinforces the importance of a One Health approach to better understand RVA evolution, transmission pathways, and zoonotic risk, particularly in countries recognized as hotspots for pathogen diversity, such as Brazil. From a surveillance perspective, uncommon genotype constellations such as G3P[3] may be underrecognized in routine diagnostic settings, emphasizing the need to integrate whole-genotype sequencing into monitoring programs.

## Figures and Tables

**Table 1:** Genotype constellation of the RVA/Human-wt/BRA/IAL-R451/2011/G3P[3] strain compared with those of reference animal and animal-like human G3P[3] RVA strains. Genotypes corresponding to the typical DS-1-like, AU-1-like, and Cat97-like constellations are marked in vintage pink, light orange and pale green, respectively. Roman numerals indicate the lineage assigned to each genome segment of the strains RVA/Human-wt/BRA/IAL-R451/2011/G3P[3], RVA/Dog-wt/BRA/IAL-M202/2017/G3P[3], RVA/Dog-wt/BRA/IAL-M214/G3P[3] and RVA/Dog-wt/BRA/IAL-M414/2017/G3P[3].

Strain	Genotypes										
	VP7	VP4	VP6	VP1	VP2	VP3	NSP1	NSP2	NSP3	NSP4	NSP5
**RVA/Human-wt/BRA/IAL-R451/2011/G3P[3]**	G3-III	P[3]	I2-XX	R3-II	C2-V	M3-II	A9	N2-XXIV	T3-II	E3-II	H6-I
Brazilian canine strains											
RVA/Dog-wt/BRA/IAL-M202/2017/G3P[3]	G3-III	P[3]	I2-XX	R3-II	C2-V	M3-II	A9	N2-XXIV	T3-II	E3-II	H6-I
RVA/Dog-wt/BRA/IAL-M214/2017/G3P[3]	G3-III	P[3]	I2-XX	R3-II	C2-V	M3-II	A9	N2-XXIV	T3-II	E3-II	H6-I
RVA/Dog-wt/BRA/IAL-M414/2017/G3P[3]	G3-III	P[3]	I2-XX	R3-II	C2-V	M3-II	A9	N2-XXIV	T3-II	E3-II	H6-I
Canine strains											
RVA/Dog-tc/JPN/RS15/1982/G3P[3]	G3	P[3]	I3	R3	C2	M3	A9	N3	T3	E3	H6
RVA/Dog-tc/ITA/RV198-95/1995/G3P[3]	G3	P[3]	I3	R3	C2	M3	A9	N2	T3	E3	H6
RVA/Dog-tc/ITA/RV52-96/1996/G3P[3]	G3	P[3]	I3	R3	C2	M3	A9	N2	T3	E3	H6
RVA/Dog-tc/USA/A79-10/1979/G3P[3]	G3	P[3]	I3	R3	C2	M3	A9	N2	T3	E3	H6
RVA/Dog-tc/USA/CU-1/1982/G3P[3]	G3	P[3]	I3	R3	C2	M3	A9	N2	T3	E3	H6
RVA/Dog-tc/USA/K9/1981/G3P[3]	G3	P[3]	I3	R3	C2	M3	A9	N2	T3	E3	H6
RVA/Dog-wt/HUN/135/2012/G3P[3]	G3	P[3]	I3	R3	C3	M3	A15	N2	T3	E3	H6
RVA/Dog-tc/CHN/WH/2020/G3P[3]	G3	P[3]	I3	R3	C3	M3	A9	N2	T3	E3	H6
RVA/Dog-wt/AUS/centralwest_3/2022/G3P[3]	G3	P[3]	I3	R3	C3	M3	A9	N2	T3	E3	H6
RVA/Dog-wt/AUS/huntervalley_3/2022/G3P[3]	G3	P[3]	I3	R3	C3	M3	A9	N2	T3	E3	H6
RVA/Dog-wt/THA/CU126/2017/G3P[3]	G3	P[3]	I3	R3	C3	M3	A9	N2	T3	E3	H6
RVA/Dog-wt/THA/CU128/2017/G3P[3]	G3	P[3]	I3	R3	C3	M3	A9	N2	T3	E3	H6
RVA/Dog-wt/THA/CU132/2017/G3P[3]	G3	P[3]	I3	R3	C3	M3	A9	N2	T3	E3	H6
RVA/Dog-wt/THA/CU20139/2017/G3P[3]	G3	P[3]	I3	R3	C3	M3	A9	N2	T3	E3	H6
RVA/Dog-wt/THA/CU23379/2019/G3P[3]	G3	P[3]	I3	R3	C3	M3	A9	N2	T3	E3	H6
RVA/Dog-wt/THA/CU25012/2020/G3P[3]	G3	P[3]	I3	R3	C3	M3	A9	N2	T3	E3	H6
RVA/Dog-wt/THA/CU25170/2020/G3P[3]	G3	P[3]	I3	Rx	C3	M3	A9	N2	T3	E3	H6
Feline strains											
RVA/Cat-wt/THA/CU25045/2020/G3P[3]	G3	P[3]	I8	R3	C3	M3	A9	N3	T3	E3	H6
RVA/Cat-tc/AUS/Cat97/1984/G3P[3]	G3	P[3]	I3	R3	C2	M3	A9	N2	T3	E3	H6
RVA/Cat-tc/JPN/FRV72/1990/G3P[3]	G3	P[3]	I3	R3	C2	M3	A9	N2	T3	E3	H6
RVA/Cat-tc/JPN/FRV64/1989/G3P[3]	G3	P[3]	I3	R3	C2	M3	A9	N2	T3	E3	H6
RVA/Cat-tc/JPN/FRV73/1990/G3P[3]	G3	P[3]	I3	R3	C2	M3	A9	N2	T3	E3	H6
RVA/Cat-tc/JPN/FRV303/1993/G3P[3]	G3	P[3]	I3	R3	C2	M3	A9	N2	T3	E3	H6
RVA/Cat-tc/JPN/FRV348/1993/G3P[3]	G3	P[3]	I3	R3	C3	M3	A15	N3	T3	E3	H6
Canine/Feline-like human strains											
RVA/Human-wt/JPN/12638/2014/G3P[3]	G3	P[3]	I3	R3	C3	M3	A9	N2	T3	E3	H6
RVA/Human-wt/CHN/M2-102/2014/G3P[3]	G3	P[3]	I3	R3	C3	M3	A9	N3	T3	E3	H6
RVA/Human-wt/BRA/143/2003/G3P[3]	G3	P[3]	I3	Rx	C2	Mx	Ax	N2	T3	E3	Hx
RVA/Human-wt/IRS/Ro1845/1985/G3P[3]	G3	P[3]	I3	R3	C2	M3	A9	N2	T3	E3	H6
RVA/Human-wt/RUS/Leningrad-568/1988/G3P[3]	G3	P[3]	I2	R2	C3	M3	A9	N2	Tx	E3	H6
RVA/Human-tc/ITA/PA260-97/1997/G3P[3]	G3	P[3]	I3	R3	C3	M3	A15	N2	T3	E3	H6
RVA/Human-wt/USA/HCR3A/1984/G3P[3]	G3	P[3]	I3	R3	C2	M3	A9	N2	T3	E3	H6
RVA/Human-wt/USA/6212/2003/G3P[3]	G3	P[3]	I3	R3	C2	M3	A9	N2	T3	E3	H6
RVA/Human-wt/USA/6235/2003/G3P[3]	G3	P[3]	I2	R3	Cx	M3	A9	N2	T3	E3	H6
Wild animal strains											
RVA/Raccoon-wt/CHN/SD-MO5/2021/G3P[3]	G3	P[3]	I3	R3	C3	M3	A9	N2	Tx	E3	H6
RVA/Simian-tc/USA/RRV/1975/G3P[3]	G3	P[3]	I2	R2	C3	M3	A9	N2	T3	E3	H6
RVA/Rat-wt/ITA/Rat14/2015/G3P[3]	G3	P[3]	I1	R11	C11	M10	A22	N18	T14	E18	H13
RVA/Rat-wt/GER/KS-11-573/2011/G3P[3]	G3	P[3]	I20	R11	C11	M10	A22	N2	T14	E18	H13
RVA/Rat-wt/CHN/LQ285/2013/G3P[3]	G3	P[3]	I3	R3	C3	M3	A9	N3	T3	E3	H6
RVA/Mouse-wt/CHN/LQ6/2013/G3P[3]	G3	P[3]	I3	R3	C3	M3	A9	N3	T3	E3	Hx
RVA/Mouse-wt/CHN/LQ321/2013/G3P[3]	G3	P[3]	I3	Rx	C3	Mx	A9	N3	T3	E3	H6
RVA/Bat-wt/BRA/4754/2013/G3P[3]	G3	P[3]	Ix	Rx	Cx	Mx	Ax	Nx	T3	E3	H6
RVA/Bat-wt/ZMB/LUS1214/2012/G3P[3]	G3	P[3]	I3	R2	C2	M3	A9	N2	T3	E2	H3
RVA/Bat-wt/BGR/BB89-15/2008/G3P[3]	G3	P[3]	I3	R3	C3	M3	A9	N3	T3	E3	H6
RVA/Bat-wt/GAB/GKS-897/2009/G3P[3]	G3	P[3]	I8	R8	C5	M5	A5	N3	T5	E3	H5
RVA/Bat-wt/GAB/GKS-954/2009/G3P[3]	G3	P[3]	I16	R8	C5	M5	A5	N3	T3	E3	H5
RVA/Bat-wt/GAB/GKS-912/2009/G3P[3]	G3	P[3]	I16	R8	C5	M5	A5	N3	T3	E3	H5
RVA/Bat-wt/CHN/MSLH14/2012/G3P[3]	G3	P[3]	I8	R3	C3	M3	A9	N3	T3	E3	H6
RVA/Bat-wt/CHN/YSSK5/2015/G3P[3]	G3	P[3]	I8	R20	C2	M1	A9	N3	T3	E3	H6
RVA/Bat-wt/CHN/BSTM70/2015/G3P[3]	G3	P[3]	I8	R3	C3	M3	A29	N3	T3	E3	H6
RVA/Bat-wt/CHN/LZHP2/2015/G3P[3]	G3	P[3]	I3	R3	C3	M3	A9	N3	T3	E3	H6
Domestic animal strains											
RVA/Horse-wt/ARG/E3198/2008/G3P[3]	G3	P[3]	I3	R3	C3	M3	A9	N3	T3	E3	H6
RVA/Horse-wt/IND/ERV6/2017/G3P[3]	G3	P[3]	I8	R3	C3	M3	A9	N3	T3	E3	H6
RVA/Horse-wt/IND/ERV4/2017/G3P[3]	G3	P[3]	I8	R3	C3	M3	A9	N3	T3	E3	H6
RVA/Rabbit-wt/CHN/ZJ-F1/2020/G3P[3]	G3	P[3]	I2	R3	C3	M3	A9	N2	T1	E3	H3

## Data Availability

The nucleotide sequences have been deposited in GenBank under the accession numbers: PZ161290-PZ161300.

## Supplementary Materials

The following supporting information can be downloaded at: https://www.mdpi.com/article/10.3390/tropicalmed11060144/s1, File S1: Length and nucleotide position of each gene segment of the RVA/Human-wt/BRA/IAL-R451/2011/G3P[3] strain; Table S1: Complete list of strains included in the phylogenetic analyses of the 11 genomic segments of the RVA/Human-wt/BRA/IAL-R451/2011/G3P[3] strain.
