# Supplementary material for: Zoonotic Spillover of a Canine-like Rotavirus A G3P[3] Strain in a Brazilian Child
Source: Trop Med Infect Dis. 2026 May 26;11(6):144. doi: 10.3390/tropicalmed11060144 (PMC13307553; doi:10.3390/tropicalmed11060144)
Supplement: Supplementary file 1 [file tropicalmed-11-00144-s001.zip › File S1. Length and nucleotide position.pdf]

File S1. Length and nucleotide position of each gene segment of the RVA/Human-wt/BRA/IAL-R451/2011/G3P[3] strain.

				IAL-R451/2011	
**Gene segment**	**Size (nt)**	**Encoded protein**	**ORF position (nt)**	**Size (nt)**	**Position (nt)**
1	3,302	VP1	19-3,285	1,061	844-1,904
2	2,690	VP2	17-2,656	718	80-794
3	2,591	VP3	50-2,557	466	2,125-2,590
4	2,362	VP4	10-2,337	762	77-838
5	1,611	NSP1	11-1,492	1,497	7-1,503
6	1,356	VP6	24-1,217	1,308	28-1,329
7	1,104	NSP3	16-948	953	26-1,020
8	1,059	NSP2	30-983	971	47-1,017
9	1,062	VP7	49-1,029	992	37-1,028
10	751	NSP4	42-569	684	26-709
11	667	NSP5/6	5-598	667	1-667
**Total genome**	**18,474**	**-**	**-**	**10,079**	**-**
