# Supplementary material for: Zoonotic Spillover of a Canine-like Rotavirus A G3P[3] Strain in a Brazilian Child
Source: Trop Med Infect Dis. 2026 May 26;11(6):144. doi: 10.3390/tropicalmed11060144 (PMC13307553; doi:10.3390/tropicalmed11060144)
Supplement: Supplementary file 1 [file tropicalmed-11-00144-s001.zip › Supplementary Table S1.pdf]

Supplementary Table S1. Complete list of strains included in the phylogenetic analyses of the 11 genomic segments of the RVA/Human-wt/BRA/IAL-R451/2011/G3P[3] strain.

Segment	Genotype	Lineage	Strains included
VP7	G3	Lineage I	E2451, CC425, PB1048,AU-1, R142, RotaTeq-WI78-8, 107E1B, 3000503692, TO-243, TO-231, TO-154, RS20345, CMH054, RJ10911, R1267, IAL-R2758
		Lineage II	RV52-96, PA260-97, N5, RRV
		Lineage III	As shown in Fig. 1A
		Lineage IV	BE5028, 30-96, B4106
		Lineage V	CMP29, CP-1
		Lineage VI	39128, 31128, 32128, 77132, 17128, 06123, 13128, 1282, 14V08023, 4099, 0988, 6572, 8973, 11V10001, 15V08513, 13V09375, CM3, CVH3, 12V08472, 7151, 5283, 65131, 91128, 3855, 2059, 7656, 9051, 35111, 03V04954, 52634, BI, E8702, E6094-3, E9402, E4394, E379, E384, E152, E30, E5749-4, E4323, E394, E1460-2, E5975, E6000, E1668, E405, E9562-3, H-2, 4698G5, 4775G7, ERV316, KY66, KY69, KY-91, KY48, KY16, KY80-2, No.97, KY80-1, No.101, KY81-1, No.7,KY81-2, No.35, 4616G11, 4766G3, No.21, H2, No.204, JE29, No.146, No.60, Nasuno, JE75, JE97, HH-22
		Lineage VII	J63, Erv80, MSLH14
		Lineage VIII	QUI-135-F1, 1A3739, CMH222
		Lineage IX	IAL-R751, LVCA29653, 105-606-D039, ERN8187, GO-MR, AM-16-67, IS1078, GER34-16, SOEP128, D388, 3000503706, SS75915299, SKT-281, 3000390639, 15R429
VP4	P[3]	No identified lineages	As shown in Fig. 1B
VP6	I2	Lineage I	DS-1, 83A001, CK20001
		Lineage II	L26, AK26, PAI11
		Lineage III	KUN, 80SR001
		Lineage IV	AU605, TB-Chen, AU109, HSE005
		Lineage V	0308, BID1B9, BID1B8, MW5-039, MW5-064, OP5-009, MRC-DPRU1673, MRC-DPRU1280-05, MRC-DPRU1362, MRC-DPRU81, RCH041, CK20052, TO-251, IP-447, TO-075, 2009727104, 2009727045, 2009727137, IAL-R2597, IAL-R2601, JP11105, MS11142, 1040SR, US8908, US5139, Nov05-202, RV161, CK20016, MRC-DPRU3180, GHNAV483, GHNAV482, MRC-DPRU2201, MRC-DPRU5625, MRC-DPRU5594, MRC-DPRU5608, MRC-CPRU5615, MRC-DPRU1818, B1711, MRC-DPRU618, IAL-RN377, IAL-R2437, MRC-DPRU2206, KDH1968, NS17-A1301, 3001607814, IAL-R465, IAL-R52, LVCA30412, 3203WC, CAR91, SE19300, IAL-RN77996, Kol-065, RV1316, RV0903, LB1562, RV11, TO-228, QUI-164-F1, 2A3847, IAL-R86, TO-002, Nov09-D2, C-421, QUI-60-F1, IP-218 MG, mcs72,

			GHPL1989, WAPC703, CK20051, IRID-3271, RV09, CM-0019, RV0904, CM-1261, NS17-A1053, Nov10-N407, Nov09-KZ, 15898, 16101, ES16238, IAL-R1228, BID2DE, UB-432, CMH028, SA066, IAL-R193, RVN1149, IAL-R558, IAL-R3172, D388, IAL-R3122, IAL-R594, LVCA30388, IAL-R751, SS98242319, DRC88, DRC86, Ph158, GR10924
		Lineage VI	HP113, HP140, PML1965, Ghan-113, GH019-08, MW2-026, MW2-181
		Lineage VII	NT0082, PCB-79
		Lineage VIII	As shown in Fig. 1C
		Lineage IX	2371WC, MRC-DPRU2053, M0084, ma31, A75, 1604, RC-18-08, Mali-039, B10925, ORVR762, BEF06018
		Lineage X	VU11-12-193, VU11-12-53, HKD0825, RotaTeq-WI78-8, WC3, Sz-ET1009, Sz-LG0305, CK20039, B7711, BRV105, JL310083, HLJ8002, HLJ8012, NCDV, ERV2, C73, SR338, Nov10-N397, PA169, P343, OH4, OH-4, KKK3, MRC-DPRU447, Se584, B223, DQ-75, 2012841174, 1442, 4426, Sun9, SR294, GA471, S19, 88977, 111-05-27, D38-14, RRVAL232, Crassostrea gigas-BsAs, R-22, KJ19-2, KJ9-1, MVS-BRV1290xUK, BRV-KC-1xUK, MVS-BRV4
		Lineage XI	N83, 69M
		Lineage XII	30-96, B4106, BE5028
		Lineage XIII	0040, SA11-5S, Dai-10
		Lineage XIV	KF17, D205, BA222, Hun5
		Lineage XV	E9314-5, JE84, EqRV-SA1, BI
		Lineage XVI	61A, A44
		Lineage XVII	1603, MRC-DPRU3005, MRC-DPRU3878
		Lineage XVIII	GO34, MP B48, RUBV319
		Lineage XIX	A5-10, A5-13
		Lineage XX	As shown in Fig. 1C
VP1	R3	Lineage I	1093A, 1037A, 1487SR, 1135A, 942A, 1471SR, PE18974, IAL-R566, IAL-R870, IAL-R1151, PE15776, 74IPN, 52127, 55018, 56010, T152, CAU14-1-262, R11-035, To16-11, CU128, CU126, CU132, CU20139, WH, SD-MO5, CU25012, 12638, huntervalley_4, huntervalley_3, centralwest_3, SD-MO3, CU365-KK, 1256A, 1257A, 1701SR, 1259A, 1815S, FRV348, 135, PA260-97, AU1115, Mie20120016f, Mie20120003f, FRV-1, AU-1, AU938, R138, R142, R57, R55, R47
		Lineage II	As shown in Fig. 1D
		Lineage III	Z3171, ZJ-F1, JS-NATF2, N5, K8, WZ606, FRV384
		Lineage IV	WC179, RA116, RA108
		Lineage V	LQ285, LQ6, LQ269, WZ101, 11D034-1, LZHP2, KLF2230, L621, E2451

		Lineage VI	BB89-15, BR89-60, Rhi fer
		Lineage VII	TUCH, D2213150, 3000640724, 2012841174, MS2015-1-0001, Pool-311, MYAS33, CHYNC82, CMH-S015-19, CU25045, B5662, CMH079
		Lineage VIII	As shown in Fig. 1D
VP2	C2	Lineage I	83A001, CK20001, DS-1
		Lineage II	AK26, PAI11, D205, 1076, L26
		Lineage III	KUN, 80SR001
		Lineage IV non-a	TB-Chen, AU109
		Lineage IVa	Ghan-002, MRC-DPRU2206, Ghan-148, UFS-NGS-MRC-DPRU1971, GHDC514, UFS-NGS-MRC-DPRU1973, Ghan-105, Ghan-052, F01498, MRC-DPRU3165, MRC-DPRU2344, Ghan-013, MRC-DPRU228, MRC-DPRU5164, PA459, IAL-R465, IAL-R95, MRC-DPRU3046, G2P4-2-40, BA366, MA228, BA346, ES298, MRC-DPRU4680, MRC-DPRU3032, BID1BI, Mali-071, Mali-069, Ghan-053, Mali-035, Mali-045, Ghan-009, tGd28, MRC-DPRU3180, Mali-074, Mali-028, Ghan-108, MA104, G2P4-1, 3001607810, 3001607819, 3001607822, 3001607809, 3001607620, 3001607611, 3001607594, 3001607621, 3001607626, 3994, 3001607671, 3001607722, 3001607144, PML1965, MRC-DPRU2201, MRC-DPRU5594, KDH1968, MUL-13-427, STM415, 4009, 3000592598, 3000354444, MUL-13-166, MRC-DPRU5631, MRC-DPRU3199, MUL-12-104, MRC-DPRU5124, MRC-DPRU4960, 2014738139, BE83, MUL-13-308, IAL-R86, MUL-13-157, 2371WC, MRC-DPRU2053, IAL-R2437, IAL-RN377, MW1-860, MW2-026, MW1-023, MUL-13-204, MW2-924, MW2-489, MW2-624, 1473, MW2-1238, MW2-1246, QEC257, MRC-DPRU2131, QOP002, MRC-DPRU1818, B1711, MRC-DPRU618, UFS-NGS-MRC-DPRU580, UFS-NGS-MRC-DPRU531, 3203WC, IAL-R126, IAL-R1228, BID2DE, CK20051, NIV1416591, BID11D, BID124, BIDB9, S120088, Fuzhou23-33, CCH764, CAU11-04, IAL-R2597, IAL-R2601, DRC88, IAL-R52, UR14-10, IAL-R3165, RVN1149, PCB-84, D388, KN093, 16020_92, 16020_80, EnRo2275, STM008, KN141, KN095, KN076, KN118, IAL-R558, IAL-R193, SOEP156, S13-30, S13-45, RVA-U4, To17-02, Tokyo18-36, STM147, PA213, PA227, SS61720845, SS61921417, SS96217158, IAL-R3172, Tokyo18-25, SS98242319, NS17-07, IAL-R608, IAL-R751, D009617, SU17-03, SU17-02, SU17-04, SU17-15, NS17-02, TA17-02, TA17-01, SU17-20, SU17-13, SU17-07, TA17-08, TA17-04, TA17-03, SU17-24, SU17-21, SU17-18, SU17-16, TA17-06, SU17-14
		Lineage V	As shown in Fig. 1E
		Lineage VI	PR457, Ghan-149, Ghan-113, GH019-08
		Lineage VII	DQ-75, BP1062
		Lineage VIII	CC0812-1, Lamb-NT, LLR, XL
		Lineage IX	BA222, PAH136, Hun5

		Lineage X	Ro8059, NCDV, RF, 88977, OH-4, Dai-10
		Lineage XI	A64, 69M, KF17, OVR762
		Lineage XII	PAI58, 17237, Se584, PTRV, O´Agent
		Lineage XIII	FI-14, H2, HH-22
		Lineage XIV	PA169, MRC-DPRU1604
VP3	M3	Lineage I	FRV317, FRV384, K8, R70, R57, R138, R142, AU1115, AU-1, FRV-1, AU938, Mie20120016f, Mie20120013f
		Lineage II	As shown in Fig. 1F
		Lineage III	Leningrad-568, RRV, N5, E3198
		Lineage IV	L621, KFL2230, E2451, To16-11
		Lineage V	CU25012, CU25170, WH, CU23379, CU126, LUS12-14, centralwest_3, huntervalley_1, FRV348, RV52-96, PA260-97, RV198-95, 135, 55018, 56010, 52127, PE15776, IAL-R1151, T152, 74IPN, 985A, 1093A, 1471SR, 1037A, 12638, CU20139, SD-MO5, CU365-KK, 1709SR, 1257A, 1701SR, 1702SR, 1259A, 1256A
		Lineage VI	TUCH, D2213150
		Lineage VII	BE5028, 36-9, B4106, 30-96
		Lineage VIII	JE97, JE84, No.32, HO-5, No.28, FI23, 04V2024, EqRV-SA1, JJ01, H2, BI, 03V04954, E4040, E30, E403
		Lineage IX	As shown in Fig. 1F
		Lineage X	Rhi_fer, Rhi_hip, ZJ-F1, HB01
		Lineage XI	WZ101, LQ269, LQ6, LQ315, LQ285
		Lineage XII	As shown in Fig. 1F
NSP1	A9	No identified lineages	As shown in Fig. 1G
NSP2	N2	Lineage I	CK20001, DS-1, 83A001
		Lineage II	The original classification proposed by Agbemabiese et al [40] did not describe a Lineage II
		Lineage III	KUN, 80SR001
		Lineage IV	TB-Chen, R49, HSE005, NSC209, NSC212, NSC206
		Lineage V	IAL-RN373, IAL-R2404, IAL-RN361, MRC-DPRU5164, DRC88, MRC-DPRU1818, SE19300, SC19868, MW1-131, RV161, B110005, 2A3618, 2A3406, 2A3762, 2A3291, 1A2792, 1A3320, 2A3292, 2A3257, 2A3847, 1A3178, 1A3186, QUI-164-F1, QUI-158-F2, QUI-157-F1, QUI-130-F2, QUI-73-F3, QUI-73-F2, 1A2604, 2A3112, 1A3385, IAL-R86, QUI-154-F1, QUI-74-F2, RS13086, MA14286, RS15851, MA19557, 18664CE, 18649CE, RJ12225, SE13801, MS11142, IAL-R126, ES16238, 18838CE, 18773CE, RJ17745, QUI-36-F1, 18807CE, 18679CE, QUI-60-F1, QUI-59-F1, IAL-R95, IAL-R52, IAL-R465, IAL-50, IAL-R3594, IAL-R330, IAL-R530, IAL-R608, IAL-R751, IAL-R645, IAL-R3172, IAL-R3165, IAL-R3123,

			IAL-R3122, D388, MA125, CO017
		Lineage VI	04V2024, E30, HH-22
		Lineage VII	GH019-08, Ghan-113
		Lineage VIII	12034, LLR, CC0812-1, AU109, NT0578
		Lineage IX	PAK419, Ghan-059, CM-423, CM-0002, Hun5, CMC_00022, MRC-DPRU447
		Lineage X	S19, MRC-DPRU1862, AS970, Egy3399, Amasya-1, B10925, BP1879, OVR762, ma31, 88977, 182-02, SS65, 2009727137, 2009727045, GER1H-09, IAL-R2597, 2009727104, 2009727103, IAL-R2598
		Lineage XI	2371WC, Mali-039, Mali-048, MRC-DPRU2053
		Lineage XII	A64, 69M, V585, RC-18-08
		Lineage XIII	PTRV, Tokyo12-1375, 14-02218-2, Leningrad-568, RRV, Bov4, Bov7, GB12-22, 2012841174, DQ-75, KM3, SR294, 2009726790, MN41364, 2012741499, RF, NCDV, BRV105, WC3, UFS-NGS-MRC-DPRU442, SX-BrB-9, CK20039, Sz-ET1009, Rotateq-WI79-4, VU12-13-176, VU12-13-42, VU12-13-177, HKD825, B8019
		Lineage XIV	O´Agent, SA11-N2
		Lineage XV	B383, LVMS1788, Mali-072, LVMS3053, LVMS2625, 492SR, MRC-DPRU3010, 1442, MRC-DPRU3005, MPT-307, PCB-85, UR14-14, RVN1149, IAL-R558
		Lineage XVI	CU126, CU128, CU132, CU20139, huntervalley_1, huntervalley_3, 12638, LUS12-14, CU23379, HB01, 135, RV52-96, RV198-95, PA260-97, KF17, Nov07-2253
		Lineage XVII	ERN5162, 12US1134, PAI58, B4106, 30-96
		Lineage XVIII	RCH272, MG6
		Lineage XIX	C75, Chubut
		Lineage XX	CH-3, BI
		Lineage XXI	KJ9-1, KJ-69, KJ9-2, NT0082, 24, AH1207, 3000015004, Rio_Negro, SI-B17, Dai-10, KK3, Tottori-SG, AH1041
		Lineage XXII	FRV72, FRV64, FRV73, Ro1845, CU-1, Cat97, A79-10, K9, HCR3A, FRV303, 6212, 143, 6235
		Lineage XXIII	1603, A44, 61A
		Lineage XXIV	As shown in Fig. 1H
		Lineage XXV	As shown in Fig. 1H
NSP3	T3	Lineage I	3000381850, VE5566, CU33427, BA222, KF17, Mie2012001, AM582, GER29-14, ERN5162, K8, RV10109, FRV-1, FRV384, FRV317, PA307, AU12-2-51, E2451, ma19030-10, rj14055, AU938, AU-1, AU1115, R55, R57, R138, R47, R142
		Lineage II	As shown in Fig. 1I
		Lineage III	IAL-R1151, IAL-R870, PE18974, T152, 74IPN, PE15776, 52127, 55018, 1037A, IAL-R566, FRV348,

			CU20139, SD-MO5, LUS12-14, CU23379, 12638, centralwest_3, huntervalley_1, CU132, CU128, CU126
		Lineage IV	S18CXBatR24, rab1404, E3198, BB89-15, RRV, MHSMC16, RAGXC17, To16-11, L621, ROMA116, CU365-KK, 1701SR, 1259A
		Lineage V	MSLH14, MS2015-1-0001
		Lineage VI	GKS-941, GKS-954, GKS-934, ERV6, ERV4
		Lineage VII	B4684, MYAS33, YSSK5, M2-102, LZHP2, D22131, 09US7118, TUCH, SA44, E403, E4040, E30, 1A3739, QUI-15-F11, QUI-135-F1, QUI-67-F3, QUI-140-F1, QUI-35-F5, QUI-146-F1, QUI-139-F1
		Lineage VIII	No.35, No.97, No.101, No.71, 03V04954, No.79, No.30, No.32, JE84, FI23, No.67, No.36
		Lineage IX	GH018-08, GH019-08
NSP4	E3	Lineage I	NN347-22, 20200920, NN2924-21, NN2853-21, 20200718, NN2505-15, PG05, 20210919, Mie20120017f, K17, 20171105, CAU12-2, NN496-16, NN2885-21, NN2748-18, 2020999, Fuzhou22-38, BJ-F2086, NN21-23, Fuzhou23-3, SC24-002ds, RV10109, IP-218, Mie20120016f, FRV1, AU-1, AU938, E2451, AU115, R47, R55, R57, R142, R138, 56010, 55018
		Lineage II	As shown in Fig. 1J
		Lineage III	TUCH, Z3171, R3265, rab1404, M2-102, LZHP2, BatRVA322, BB89-15, E3198, R2, N5, RRV, leningrad-568, K8, FRV317, FRV381, FRV384, CAU14-1-262, R11-035, 20190906, SARN53, ROMA116, CMH120, L621, 1709SR, 1256A, 1701SR, 1702SR, 1257A
		Lineage IV	RS15, PE18974, IAL-R870, IAL-R1151, 1093A, 1157A, y942ASR06, 985A, 1487SR, 1135A, 037A, 152, IAL-R566, UB-144_MG, PE15776, 74IPN, 52127
		Lineage V	huntervalley_3, huntervalley_4, CU126, SD-MO5, CU23379, CU20139, 12638, 135, FRV348, R1486, PA260-97, RV52-96
		Lineage VI	MSLH14, ERV4, ERV6, MS2015-1-0001, To16-11, MRC-DPRU295
		Lineage VII	YSSK5, BSTM70
		Lineage VIII	MYAS33, CMH079
		Lineage IX	1A3739, QUI-67-F3, QUI-15-F11, QUI-35-F5, QUI-135-F1, QUI-146-F1, QUI-140-F1
		Lineage X	Alp5403, AlpH39, JCRQ, YD, ADR, FRNM, AlpHSN, AlpSA25, SL10, LAO, TDNM, SA44, AlpS005
NSP5	H6	Lineage I	As shown in Fig. 1K
		Lineage II	FRV348, FRV303, T152, 74IPN, 56010, PE15776, Py942ASR06, 1037A, B-150, PE18974, IAL-R870, IAL-R566, UB-144MG
		Lineage III	D2213150, RRV, Leningrad-568
		Lineage IV	12638, huntervalley_1, centralwest_3, CU20139, CU23379, SD-MO5, CU126, CU25012, WH
		Lineage V	E2451, To16-11, R11-035, CAU14-1-262, CU365-KK, 1257A, 1256A, 1701SR, L621, 1709SR, SARN53
		Lineage VI	MYAS33, YSSK5, LZHP2, MSLH14, MS2015-1-0001

	Lineage VII	Alp22, Alp5403, SA44, AlpSA28, SL10, AlpHSN, AlpH24, Alp11A, P10, P6, LAO, JCRQ, MA24, MA30
	Lineage VIII	QUI-139-F1, QUI-15-F1, QUI-146-F1, QUI-67-F3, 1A3739, QUI-35-F5, QUI-135-F1, QUI-140-F1
